# Evaluating acoustic representations and normalization for rhoticity classification in children with speech sound disorders

**DOI:** 10.1121/10.0024632

**Published:** 2024-02-01

**Authors:** Nina R. Benway, Jonathan L. Preston, Asif Salekin, Elaine Hitchcock, Tara McAllister

**Affiliations:** 1 Communication Sciences & Disorders, Syracuse University, Syracuse, New York 13244, USA; 2 Electrical and Computer Engineering, University of Maryland, College Park, Maryland 20742, USA; 3 Electrical Engineering and Computer Science, Syracuse University, Syracuse, New York 13244, USA; 4 Communication Sciences & Disorders, Montclair State University, Montclair, New Jersey 07043, USA; 5 Communicative Sciences & Disorders, New York University, New York, New York 10007, USA

## Abstract

The effects of different acoustic representations and normalizations were compared for classifiers predicting perception of children’s rhotic versus derhotic /ɹ/. Formant and Mel frequency cepstral coefficient (MFCC) representations for 350 speakers were z-standardized, either relative to values in the same utterance or age-and-sex data for typical /ɹ/. Statistical modeling indicated age-and-sex normalization significantly increased classifier performances. Clinically interpretable formants performed similarly to MFCCs and were endorsed for deep neural network engineering, achieving mean test-participant-specific F1-score = 0.81 after personalization and replication (*σ_x_* = 0.10, med = 0.83, *n* = 48). Shapley additive explanations analysis indicated the third formant most influenced fully rhotic predictions.

## 1. Introduction

Validated speech analysis algorithms could someday mitigate access barriers to sufficiently intense therapy for those with speech sound production difficulties by increasing treatment intensity through at-home practice with clinical grade feedback that is customized for individual learners based on their speech-language pathologist’s perceptual judgment.^1^ However, systematic review has highlighted related hindrances to the development of efficacious clinical speech technologies to date, particularly for child speakers.^2–4^ For those with residual speech sound disorders (RSSD, whose errors continue past eight years of age) our recent contribution, the PERCEPT-R Corpus,^5^ may offset one hindrance: data scarcity for child speakers. This current contribution addresses a second hindrance: the need for child speech analysis systems to offer sufficient documentation of technical development.^2^

This study focuses on the classification of fully rhotic vs derhotic /ɹ/, the most frequently impacted phoneme among American English speakers with RSSD.^6^ Recent studies highlight development barriers for clinically replicable mispronunciation detection systems in children with speech sound disorders. Li *et al.*^7^ assessed support vector machine classification accuracy relative to expert-listener ground truth for correct and misarticulated /ɑɹ/ syllables for 23 individuals with RSSD and 17 children with typical speech. The authors quote lab-tested percent accuracies exceeding 89% for the classification of ultrasound representations of imitated /ɑɹ/. The clinical replicability of this result, however, is presently limited by test set performance reflecting only one syllable context and including individuals without speech disorders. Separately, Ribeiro *et al.*^8^ found that Mel frequency cepstral coefficient (MFCC) representations outperformed ultrasound image representation in the case of /ɹ/ classification from deep neural network goodness of pronunciation scores, and reanalysis of eight test speakers with speech sound disorder in Table 6 of Ribeiro *et al.* suggests a test-participant-specific F1-score^22^ = 0.65 (*σ_x_* = 0.23). However, the authors only analyzed clearly correct/incorrect ground truths for their analysis (i.e., excluded ambiguously-rated /ɹ/s for which multiple raters did not agree on rhoticity). Because /ɹ/ attempts evoking rater perpetual disagreement are expected during speech therapy,^9^ the reported performance of a model without ambiguously-rated /ɹ/ may not generalize to real-world clinical use cases. This limits the clinical replicability of these results.

This study extends these foundations with the following performance and replicability contributions. First, ultrasound systems are not clinically common, so this study employs audio classification. Additionally, although age-and-sex normalization may be particularly important for child speech, previous rhotic classification studies do not appear to have normalized acoustic representations about age and sex. Our contribution quantifies the effect of this normalization, which is likely important because acoustic representations interact not only with vocal tract configuration but also with vocal tract size. It may be that the distribution of fully rhotic acoustic representation from a younger child with a smaller vocal tract overlaps with the distribution of derhotic acoustic representation from an older adolescent with a larger vocal tract. This can be seen in the Lee *et al.*^10^ formant dataset, in which the third quartile of F3–F2 distance (salient to rhotic perception^11^) in fully rhotic utterances from younger speakers approaches the first quartile of *neutral* vocal tract values in older speakers. Such overlap might complicate classification in child feature sets not normalized for age and sex. Indeed, age-and-sex normalization of F3–F2 difference for children aged from 6 to 12 improves modeling of listener perception of /ɹ/.^11^ A related contribution herein is that we make our MFCC-based normative values publicly available on our Open Science Framework page (https://osf.io/nqzd9/), to complement existing formant-based values.^10^

Second, the work of Berisha *et al.*^12^ underscores that clinical speech technology development must emphasize clinical replicability. We show that the performance of clinically interpretable formant representations is statistically similar to commonly used MFCC representations. One rationale supporting the ubiquity of MFCC-based classifiers is that MFCC representations summarize the breadth of a power spectrum while emphasizing perceptually relevant frequencies. However, decades of acoustic phonetics literature have quantified fully rhotic and derhotic /ɹ/ using formants that model the center frequencies and bandwidths that are emphasized in the power spectrum due to vocal tract configuration. Specifically for American English /ɹ/, the third formant (F3) encodes perceptually relevant information about rhotic or derhotic vocal tract configuration,^13,14^ making formant-based representations a compelling candidate for clinically interpretable rhoticity classifiers.

Our contribution also emphasizes clinical replicability through several experimental design steps. Whereas previous work on child speech classification has discarded ambiguous utterances^8^ and measured classifier performance on typical speakers with low clinical relevance,^7^ we retain utterances with ambiguous ratings and constrain typical speakers to the training set. We also contend that automated treatment is only ethical for individuals with RSSD who can occasionally produce fully rhotic exemplars (i.e., those who are clinically *stimulable*; fully rhotic/derhotic utterance proportion > 0.33), and it may be that these stimulable speakers produce /ɹ/ that are most difficulty to reliably rate. These decisions may ultimately degrade reported performance by selecting the most ambiguous utterances and speakers for validation and testing, but we show that a classifier designed in this way can perform above a 0.8 standard for clinical utility informed by the threshold of McKechnie *et al.*^2^

## 2. Method

We used a supervised machine learning framework to perform binary classification of fully rhotic (i.e., clinically correct) /ɹ/ vs derhotic (i.e., clinically incorrect) /ɹ/ in an American English dataset labeled with listeners’ perceptual judgments. Specific steps were taken to emphasize replicability and a model/replicability card is available at https://osf.io/nqzd9/.

### 2.1 Corpus data and experimental datasets

Speech data come from the PERCEPT-R v2.2.2 Corpus, an extension of the open-access PERCEPT-R Corpus v2.2.2p^5^ also including participants who provided permission for private use. Binary class labels were derived from the average of multi-listener perceptual ratings of rhoticity in words and short phrases, with 0.66 serving as the floor for class 1 (the “correct”/fully rhotic class; vs class 0, the “incorrect”/derhotic class) to reflect that there is often not a full agreement between expert raters in the context of RSSD.^15^

As shown in Table 1, 350 corpus participants were split into training, validation, and test datasets, containing ~70%, 15%, and 15% of utterances. To maximize external validity to clinical use cases, all typical speakers were excluded from the validation and test sets. Stimulable participants with RSSD were selected for validation and test age-and-sex stratified random allocation without replacement, to permit *post hoc* exploration relative to these covariates. The PERCEPT-R Corpus participant IDs assigned to each dataset are available https://osf.io/nqzd9/. No participant appeared in more than one dataset.

In addition to selecting stimulable participants, we downsampled PERCEPT-R Corpus data. Training set classes were balanced 1:1. Validation and test sets were balanced by simulating the 2:1 correct /ɹ/: incorrect /ɹ/ ratio expected in clinal use, based on reanalysis of 229 934 previous therapy practice trials with stimulable participants using a clinician-driven version of the computerized intervention that we intended to automate with this classifier.

### 2.2 Feature extraction

#### 2.2.1 Estimation of rhotic interval timestamps

Gaussian Mixture Modeling and Hidden Markov Modeling (GMM-HMM) forced alignment estimates of rhotic timestamps were made using the Montreal Forced Aligner.^16^ Each rhotic-associated interval was expanded with a 10 ms buffer to counteract edge loss of samples. To assess automated performance, no alignments were hand corrected.

#### 2.2.2 Formant estimation

Formants were extracted from these rhotic-associated intervals using the Praat “To Formants: Robust” algorithm. Five formants were estimated from 5 ms Gaussian-like windows, with a 5 ms step between analysis frame centers and pre-emphasis above 50 Hz. Robust refinement of formant estimates used default settings: selected weighting of samples started ± 1.5 standard deviations from the mean and stopped after five iterations or when the relative change in variance < 1 × 10^−5^. Praat function calls were facilitated with Python using the Parselmouth API.

Linear predictive coding coefficients were calculated using the Burg algorithm. We customized the *Formant Ceiling* for each speaker, important for child speech analysis.^17^ For training set participants, we optimized, through grid search,^18^ the *Formant Ceiling* that reduced regression residuals for candidate formant estimates. In the validation and test subsets, participant-average *Formant Ceiling* values were determined manually in Praat as done by Benway *et al.*^9^ to mimic the intended clinical workflow.

These *Formant Ceiling* values were used to extract formant estimates and two formant transformations (F3–F2 distance and F3–F2 deltas) from the GMM-HMM identified rhotic-associated intervals. No formant estimates were hand-corrected. The time series for each rhotic-associated interval was represented by a 3 D NumPy array of the shape (5 formants and transforms * 10 time windows * 8 summary statistics: median, mean, standard deviation, minimum, maximum, variance, skewness, and kurtosis). Shallow neural networks only processed the median value at each time window for each feature.

#### 2.2.3 MFCC estimation

MFCCs were generated in analogous fashion to formants, except with a function call to the Praat “To MFCCs” algorithm in place of the formant estimation function call. Default filter bank parameters were used; there were no speaker-specific settings. Window length and time step were identical to that which was previously noted. Thirteen MFCCs were computed with default filter bank parameters, yielding a three-dimensional (3D) array [13 MFCCs * 10 time windows * 8 summary statistics]. Shallow neural networks only processed the median value at each time window for each feature.

### 2.3 Feature normalization

We created two feature sets each for formant and MFCC representation: one feature set normalized relative to age-and-sex-specific values for correct /ɹ/ (*age-and-sex normalized* condition) and one normalized relative to the magnitude of an utterance’s own acoustic representation (*utterance-normalized* condition). For age-and-sex normalization of formants, each feature was z-standardized according to the nearest age-and-sex-matched mean for typical /ɹ/ observed by Lee *et al.*^10^ To our knowledge, no normative values exist for child rhotic MFCCs; therefore, we extracted age means and standard deviations of fully rhotic MFCCs from PERCEPT v2.2.2. These data only include training set speakers to minimize data leakage and are publicly available at https://osf.io/nqzd9/. For the utterance-normalized condition, each feature in the formant or MFCC representation was z-standardized according to the mean and standard deviation of the distribution from the self-same utterance. In both normalization conditions, the F3–F2 delta transforms underwent linear conversion for scaling between −10 and +10.

### 2.4 Classifier training for experimental comparison of candidate feature sets

We employed random forest classifiers and stochastic gradient descent classifiers to illustrate the effect of age-and-sex normalization and acoustic representation in node-based and function-based architectures allowing for warm-start retraining. The summary outcome measure was the mean F1-score, grouped by test set participant as clinical potential likely reflects speaker-level performance. Hyperparameter search was completed with 50 Optuna trials, with the goal of finding the hyperparameters that maximized participant-specific mean F1-score in the validation set. Details on hyperparameter tuning, including loss functions, are available at https://osf.io/nqzd9/.

These *out-of-the-box* tuned speaker-independent classifiers were used for inference with yet-unseen speakers in the test set. Then, to reflect the customization possible in a clinical setting, the candidate algorithms were *personalized* in a speaker-dependent fashion, with fivefold cross-validation, by adding 100 participant-specific trees to the random forest and training for ten additional epochs for support gradient descent classifiers.

### 2.5 Statistical comparison of candidate feature sets

We quantified which experimental factors imparted statistically significant effects on participant-specific average F1-score. Linear mixed-effects models estimated the main effects and all interactions of *normalization* (age-and-sex vs utterance normalization), *acoustic representation* (MFCCs vs formants), and *timepoint* (out-of-box vs personalized). Random intercepts were included for *participants* and *classifier type*. Parameters were estimated by restricted maximum likelihood.

### 2.6 Engineering a deep neural network with the endorsed feature set

**Fig. 1.**
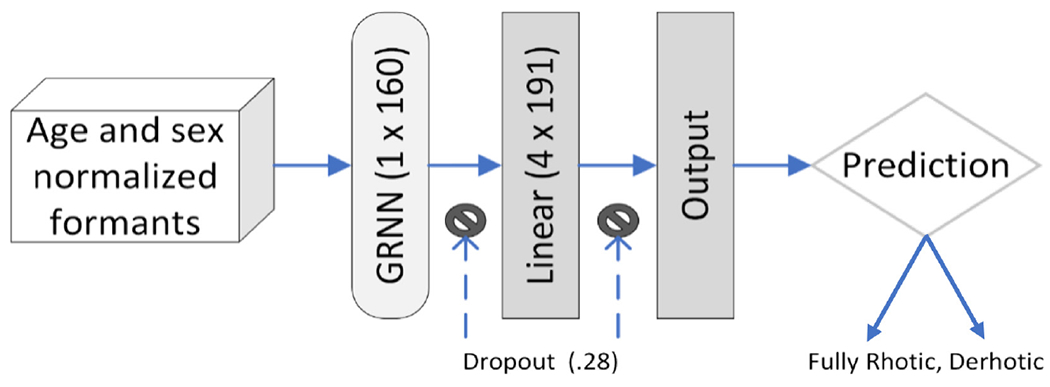
Architecture of best-performing gated recurrent neural network. The Pytorch model used binary cross-entropy loss, an RMSprob optimizer, and Hardswish neurons.

This statistical comparison was used to select features to engineer a deep neural network.^19^ The summary outcome measure was again the average test-participant-specific F1-score for the prediction of perceptual judgement of “fully rhotic” or “derhotic” /ɹ/, after personalization with fivefold within-participant cross-validation. We expected that a gated recurrent neural network (GRNN; binary cross-entropy loss) could capture temporal dependencies in long-range time-series data relevant to speech.^20^ Candidate GRNNs were trained for 25 epochs (early stopping patience = 5) and tuned with Optuna; hyperparameter details (i.e., RMSprob optimizer, Hardswish neurons) appear at https://osf.io/nqzd9/. The architecture for the best-performing deep neural network is shown in Fig. 1. As before, we tested both out-of-the-box and personalized performance. Hyperparameters details for participant-specific personalization are available at https://osf.io/nqzd9/.

#### 2.6.1 Exploring classifier bias and explainability

Last, we performed two *post hoc* analyses with the best-performing GRNN. First, a linear mixed-effects model explored the presence of age and sex effects (and interaction) on classifier mean-test-participant F1-score performance for the best-performing deep classifier. Second, Shapley additive explanations (SHAP) feature importance was analyzed with Pytorch Captum GradientShap. Because we wanted to examine how the classifier learned to differentiate ground-truth derhotic or fully rhotic productions from ambiguous input, the background set included 1973 utterances with ambiguous perceptual ratings. In contrast, the input set randomly sampled 2405 utterances with unanimous 0 or 1 ground-truth ratings. We were interested in *global importance* of the features, so the results represent SHAP estimates for each feature averaged across all utterances, time windows, and statistical representations.

## 3. Results

### 3.1 Experimental comparison of acoustic representations and normalizations

Two different shallow neural network architectures were used to compare four candidate feature sets, whose mean participant-specific F1-score performance is shown at three stages of model development in Table 2.

**Fig. 2.**
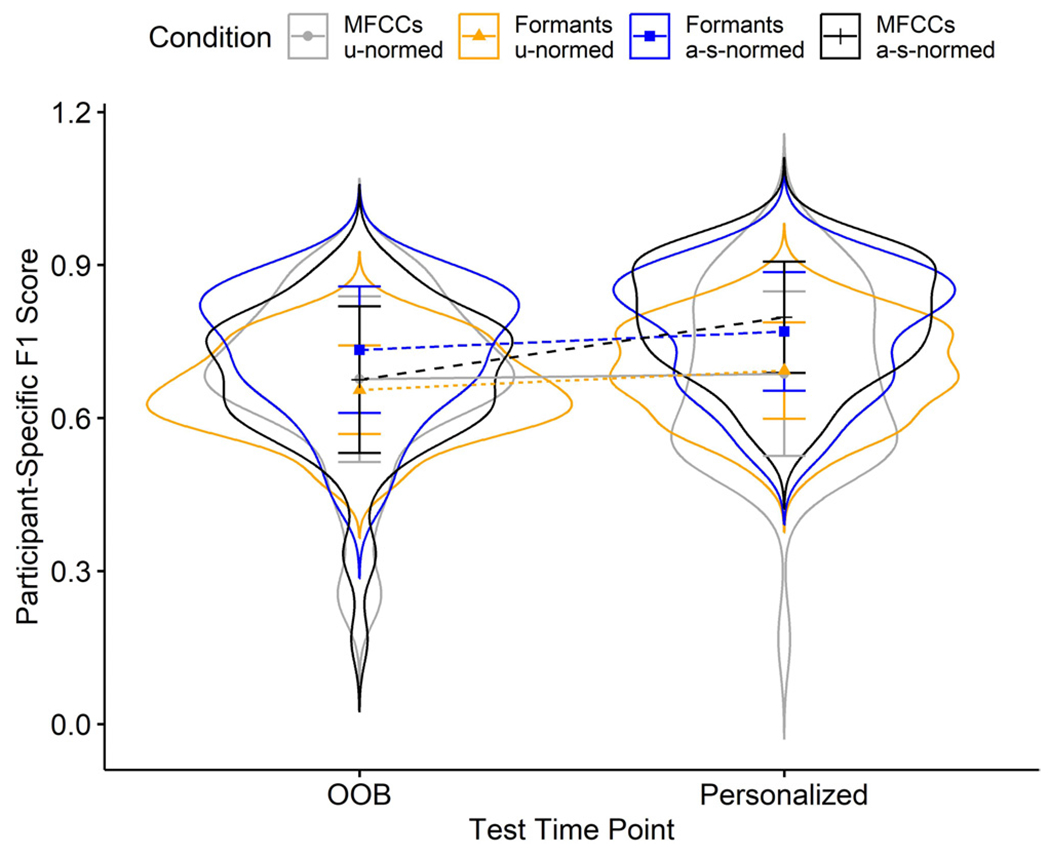
Distributions of test-participant-specific F1-scores. U-normed, utterance normalization; a-s-normed, age-and-sex normalization; OOB, out-of-box. Bars represent standard deviations.

A linear mixed model evaluating the results in Table 2, fit by restricted maximum likelihood estimation, converged with no warnings. These effects and interactions are illustrated in Fig. 2. The main effect of age-and-sex *normalization* significantly improved F1-score performance by 0.08 vs utterance-normalization (standard error, *SE* = 0.02, *df* = 382, *t* = 4.25, *p* < 0.001). The main effect of *acoustic representation* (reference level: MFCC, vs formant) did not significantly impact F1-score performance (= 0.02, *SE* = 0.02, *df* = 382, *t* = 1.13, *p* = 0.26). The main effect of *personalization* significantly improved F1-score by 0.04 vs out-of-box testing (*SE* = 0.02, *df* = 382, *t* = 2.03, *p* = 0.04). The significant representation*norming interaction indicated that, averaged across timepoints, age-sex normalization of MFCCs lowered F1-score performance by 0.08 vs age-sex normed formants (*SE* = 0.03, *df* = 382, *t* = −3.04, p < 0.01); however, the significant three-way MFCCs * age-sex * personalization interaction indicated that personalization increased age-and-sex normed MFCC performance relative to that two-way interaction by (= 0.11, *SE* = 0.04, *df* = 382, *t* = 3.06, p < 0.01).

### 3.2 Deep neural network engineering

The previous results support the use of age-and-sex normalization for deep neural network engineering, but do not provide evidence that either formant or MFCC representations systematically increased performance in this dataset. Given this, we selected formants as the acoustic representation for further engineering due to the clinical interpretability of formants relative to decades of acoustic phonetic explorations of rhoticity in typical and speech disordered speakers of American English. The best-tuned GRNN achieved a mean validation-participant-specific F1-score = 0.75 (*σ_x_* = 0.11; *n* = 22), out-of-box test-participant-specific F1-score = 0.77 (*σ_x_* = 0.13; *n* = 26), and personalized test-participant-specific F1-score = 0.83 (*σ_x_* = 0.08; *n* = 26).

Because there were relatively few test participants, we replicated the experiment by swapping the validation and test sets to increase the external validity of the results, giving a mean participant-specific F1-score for validation = 0.80 (*σ_x_* = 0.09; *n* = 26), out-of-box testing = 0.75 (*σ_x_* = 0.11; *n* = 22), and personalized testing = 0.79 (*σ_x_* = 0.09; *n* = 22). The study’s *combined* result averages the original experiment and replication; the *best* result accounts for five participants who were better predicted by the speaker-independent model. This *combined best* performance is our reported result for the engineered GRNN: average test-participant-specific F1-score = 0.81 (*σ_x_* = 0.10; med = 0.83, *n* = 48); Table 3 shows the average of participant-specific confusion matrices.

#### 3.2.1 Exploring classifier bias and explainability

A linear mixed model fit on the combined test dataset (*n* = 48) indicated that neither the fixed effects of age (= −0.020, *SE* = 0.018, *t* = −1.13, *p* = 0.265) nor sex (= −0.083, *SE* = 0.16, *t* = −0.52, *p* = 0.606) nor the age-sex interaction (= 0.013, *SE* = 0.0115, *t* = 0.904, *p* = 0.371) significantly impacted classifier performance.

Finally, SHAP estimates for age-and-sex normalized F3 and F3–F2 were positively signed, indicating the relative influence of these features when separating unanimously rated fully rhotic predictions from ambiguous input. Conversely, SHAP estimates were negatively signed for age-and-sex normalized F2, F3–F2 deltas, and F1 (i.e., first formant), indicating relative influence when differentiating unanimously rated derhotic productions from perceptually ambiguous input. Age-and-sex normalized F3 contributed the most overall importance to classifier predictions, aligning with previous acoustic studies of /ɹ/.^13,14^ Visualization of SHAP estimates appears in a figure at https://osf.io/nqzd9/.

## 4. Conclusion

This study evaluated the acoustic representations and normalization techniques that optimize the binary prediction of a listener’s perceptual judgment (i.e., fully rhotic/derhotic) of /ɹ/ in words produced by children with RSSD, finding that age-and-sex normalization statistically improved classifier accuracy vs utterance-level normalization. We interpret this as evidence that normalization can offset vocal tract differences that would otherwise confound fully rhotic and derhotic feature spaces, at least for the present age range and target phone. We hope that this line of research may someday lead to “human-in-the-loop” automated treatment that increases overall treatment intensity through at-home practice with customized, clinical grade feedback for stimulable speech sound learners.

These methods found no statistically significant difference between formant and MFCC acoustic representations. There are advantages and disadvantages to each acoustic representation. Formants are well grounded in acoustic phonetics literature and are clinically interpretable; however, extraction can be errorful^21^ and requires customization, particularly for children. Conversely, MFCCs are extracted without customization and summarize the power spectrum at multiple frequencies in a perceptually meaningful way, but are not interpretable. Given these options, we chose to base our deep classifier engineering upon (age-and-sex normalized) formant representations because of the paramount importance of clinical interpretability in the context of speech sound disorders.

We found that the best-performing GRNN classifier surpassed our participant-specific F1-score threshold for clinical utility (0.8^2^) in clinically-relevant participants. An additional strength of the present study is that we did *not* exclude speech with ambiguous ground truths. While omitting these utterances would increase the reported performance of our classifier to 0.88 out-of-the-box, such performance would not replicate clinically.

*Post hoc* exploration of age and sex effects on performance does not raise ethical concerns regarding the clinical use of the classifier relative to these demographic characteristics. It remains to be seen if age and/or sex effects are present with different speakers. A limitation of this study is that we were not able to examine the effects of ethnicity, which our ongoing work addresses. A second *post hoc* exploration with SHAP indicated that age-and-sex normalized F3 was the most influential feature on individual predictions, providing evidence that the classifier has learned previously validated, perceptually salient features for /ɹ/.

In the long term, we hope this research leads to the development of validated clinical speech technology tools. Ongoing work seeks to improve the features and designs that best separate potentially ambiguous perceptual class representations to reduce false positives, including multiclass classification techniques to capture subclinical progression toward improved productions that cannot currently be captured with binary fully rhotic/derhotic classification. Recently completed work has investigated the therapeutic value of the binary classifier developed herein, providing evidence of participant improvement for /ɹ/ in untreated words in response to an artificial intelligence (AI) assisted treatment package.

## Figures and Tables

**Table 1.** PERCEPT-R Corpus participant characteristics and utterance counts for each experimental dataset.

Subset	Total participants	Participants with speech sound disorders	Males	Females	Participant ages^a^	Number of derhotic utterances	Number of fully rhotic utterances
Training	312	193	170	142	11.4 (3.5) [6-36]	36,979	32,705
Validation	22	22	15	7	10.5 (1.7) [8-14]	5,179	9,626
Test	26	26	18	8	10.8 (3.4) [7-24]	4,849	9,183

a Age is reported as $\bar{x}$ (*σ_x_*) [range].

**Table 2.** Feature set performance, averaged across random forest and stochastic gradient descent classifier types, reported as the mean (standard deviation) of participant-specific F1-scores. The best performance at each timepoint is bolded.

Feature set	Validation performance	Test–Out-of-box	Test–Personalization
MFCCs (utterance-normalized)	0.66 (0.13)	0.68 (0.16)	0.69 (0.17)
Formants (utterance-normalized)	0.63 (0.10)	0.66 (0.09)	0.70 (0.10)
Formants (age-and-sex normalized)	**0.73 (0.11)**	**0.74 (0.13)**	0.77 (0.12)
MFCCs (age-and-sex normalized)	0.66 (0.15)	0.67 (0.14)	**0.80 (0.11)**

**Table 3.** Participant-weighted confusion matrix for *combined best*, replicated results (*n* = 48). Values are standardized by ground-truth class.

Predicted class	Ground-truth derhotic	Ground-truth fully rhotic
Derhotic	0.70	0.30
Fully rhotic	0.12	0.88

## Data Availability

The data that support the findings of this study are openly available at PhonBank (http://doi.org/10.21415/0JPJ-X403) and Open Science Framework (https://osf.io/nqzd9/).
